# The Importance of Sex in the Discovery of Colorectal Cancer Prognostic Biomarkers

**DOI:** 10.3390/ijms22031354

**Published:** 2021-01-29

**Authors:** Linnea Hases, Ahmed Ibrahim, Xinsong Chen, Yanghong Liu, Johan Hartman, Cecilia Williams

**Affiliations:** 1 Science for Life Laboratory, Department of Protein Science, KTH Royal Institute of Technology, 171 21 Solna, Sweden; linnea.pettersson@scilifelab.se (L.H.); ibrahima5050@gmail.com (A.I.); genevieveyanghong@gmail.com (Y.L.); 2 Department of Biosciences and Nutrition, Karolinska Institutet, 141 83 Huddinge, Sweden; 3 Department of Oncology and Pathology, Karolinska Institutet, 171 76 Stockholm, Sweden; xinsong.chen@ki.se (X.C.); johan.hartman@ki.se (J.H.); 4 Department of Clinical Pathology and Cytology, Karolinska University Laboratory, Södersjukhuset, 118 83 Stockholm, Sweden

**Keywords:** biomarkers, colorectal cancer, feature selection, machine learning, sex differences

## Abstract

Colorectal cancer (CRC) is the third leading cause of cancer deaths. Advances within bioinformatics, such as machine learning, can improve biomarker discovery and ultimately improve CRC survival rates. There are clear sex differences in CRC characteristics, but the impact of sex has not been considered with regards to CRC biomarkers. Our aim here was to investigate sex differences in the transcriptome of a normal colon and CRC, and between paired normal and tumor tissue. Next, we attempted to identify CRC diagnostic and prognostic biomarkers and investigate if they are sex-specific. We collected paired normal and tumor tissue, performed RNA-seq, and applied feature selection in combination with machine learning to identify the top CRC diagnostic biomarkers. We used The Cancer Genome Atlas (TCGA) data to identify sex-specific CRC diagnostic biomarkers and performed an overall survival analysis to identify sex-specific prognostic biomarkers. We found transcriptomic sex differences in both the normal colon tissue and in CRC. Forty-four of the top-ranked biomarkers were sex-specific and 20 biomarkers showed a sex-specific prognostic value. Our data show the importance of sex in the discovery of CRC biomarkers. We propose 20 sex-specific CRC prognostic biomarkers, including *ESM1*, *GUCA2A*, and *VWA2* for males and *CLDN1* and *FUT1* for females.

## 1. Introduction

Colorectal cancer (CRC) is the third leading cause of cancer deaths among both women and men in the US [1]. In Sweden, it is the second most common form of cancer in both sexes [2]. The 5-year survival rate is 91% for stage I CRC patients and 82% for stage II. However, the majority of CRC are detected at later stages with a decline in survival to 12% for stage IV CRC [3]. The poor prognosis highlights the need for new diagnostic and prognostic biomarkers to avoid CRC-related deaths. Current screening efforts include sigmoidoscopy and colonoscopy, which have been shown to significantly reduce CRC mortality. However, this association is limited to deaths from left-sided CRC [4] and participation rates remain low. Non-invasive methods using blood and stool-based tests have been proven to increase the compliance to CRC screening [5].

Identification of biomarkers, which can improve the diagnosis and disease monitoring, could significantly improve the survival rates. The advances in bioinformatics tools provide opportunities to speed up biomarker discovery and have been integrated for several cancers, including CRC [6,7]. Transcriptome studies have potential to yield large amounts of data, but the standard differential gene expression analysis has limitations. It is, for example, not performed in a multivariate setting and does not consider inter-gene relationships. Feature selection in combination with machine learning can add a new layer to the differential expression analysis and substantially improve biomarker discovery. There is also an urgent need to investigate potential sex differences in biomarker discovery. The current lack of this perspective may be one contributor to why many biomarkers fail to reach the clinic.

Sex-specific CRC recurrence and survival rates have been reported [8]. The incidence and mortality among patients over 65 years are higher for women compared to men, and the 5-year OS rate is lower for women [8]. However, the reverse is seen in pre-menopausal women [9]. Women are also more prone to right-sided CRC, which is associated with a more aggressive type compared to left-sided, more common in men [10,11]. There are also molecular sex differences where women have a higher number of B-Raf proto-oncogene, serine/threonine kinase (*BRAF*) mutations and a higher microsatellite instability (MSI) status compared to men, whereas men have a higher number of NRAS proto-oncogene, GTPase (*NRAS*) mutations [12]. Recently, we identified that mice exhibit sex differences in their colon transcriptomes [13]. Some of these differences may be related to estrogen signaling [8,14,15].

Despite the sex differences seen in CRC, most research is done without considering sex in study designs or interpretations. Sex-specific strategies for screening, prevention, and treatment should be considered in order to reduce CRC mortality. In the present study, we evaluated sex differences in the transcriptome of both non-tumor colon epithelium and CRC. Additionally, we studied sex differences in relation to diagnostic and prognostic biomarkers. Our study highlights sex differences in the normal colon, related to bile acid secretion, vitamin digestion and absorption, and in the tumor, especially related to immune response. Moreover, our study shows the importance of sex in the discovery of prognostic biomarkers. We identified 20 sex-specific prognostic biomarkers, including previously proposed biomarkers (endothelial cell-specific molecule 1/*ESM1*, guanylate cyclase activator 2A/*GUCA2A*, claudin 1/*CLDN1*) and novel ones (fucosyltransferase 1/*FUT1* and von Willebrand factor A domain containing 2/*VWA2*).

## 2. Results

### 2.1. Normal Colon and CRC Transcriptomes Exhibit Sex-Related Differences

**Figure 1:**
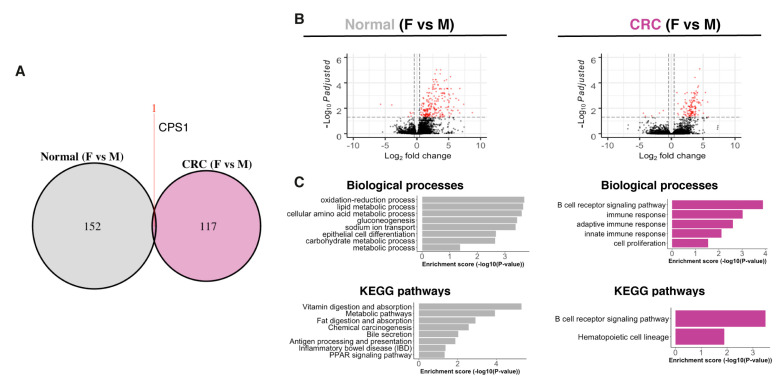
Sex differences in a normal colon and colorectal cancer (CRC) transcriptome. (**A**) Venn diagram comparing sex differences in the transcriptome of a normal colon and CRC. (**B**) Volcano plot showing differentially expressed genes (DEG) between sexes in a normal colon and CRC. (**C**) Biological process and KEGG pathway enrichment analysis of the DEG between the sexes in a normal colon and CRC.

We first validated our CRC patient cohort by exploring the expression of two well-known CRC biomarkers, early diagnostic biomarker fibronectin 1 (FN1) and prognostic biomarker cell migration inducing hyaluronidase 1 (CEMIP) [16,17]. We validated the upregulation of both *FN1* and *CEMIP* by qPCR (Figure S1A). Next, we used the RNA-seq data to identify the tumors’ molecular subtypes and compared their distribution. The distribution of the molecular subtypes in our cohort was similar to what was observed by Phipps et al. [18] (Table S1). Next, we investigated if there are sex differences in the transcriptome of normal mucosa and CRC samples. The sex differences were slightly larger in the normal mucosa compared to the CRC tissue, with 153 and 118 differentially expressed genes (DEG, with cutoff padj < 0.05, |log_2_FC| > 0.4, and fragments per kilobase of sequence per million mapped reads (FPKM) > 1), respectively (Figure 1A). The majority of the DEG was higher expressed in males compared to females (Figure 1B). Interestingly, only one gene, the mitochondrial enzyme carbamoyl-phosphate synthase 1 (*CPS1*), remained differentially expressed between the sexes in both conditions (Figure 1A). Biological process (BP) and KEGG pathway enrichment analysis revealed that the sex differences in the normal colon were related to metabolism, inflammatory bowel disease (IBD), bile secretion, epithelial cell differentiation, and PPAR signaling (Figure 1C). The sex differences in the CRC tumors were related to immune response and cell proliferation (Figure 1C). Thus, we noted clear sex differences in our cohort, both in normal and tumor tissue.

### 2.2. Transcriptomic Sex Differences Independent of Subtype and Tumor Location

Since it is well known that the sexes present differences in tumor location and characteristics [10,11,12], we investigated the distribution of tumor location and CRC molecular subtypes 1–5 based on the classification proposed by Jass in 2007 [19], which may be confounding factors in the analysis. There was no significant difference in the tumor location between the sexes (Figure S1B), which suggest that the sex differences in the transcriptomic analysis were not attributed to differences in tumor location. The females presented all subtypes whereas the males only presented subtype 3 and subtype 4 (Figure S1C). Subtype 5 clustered apart from subtypes 3 and 4 in the principal component analysis (PCA) plot (Figure S1E). In order to exclude the effect of subtype differences in the analysis, we repeated the analysis with subtypes 3 and 4 only (Figure S1C,D). The majority (75%) of the DEG remained differentially expressed between the sexes (Figure S1F–G), and the predominant pathways were still related to immune response (Figure S1H). This suggests that the sex differences in the tumor transcriptome related to immune response were not attributed to differences between molecular subtypes.

### 2.3. Paired Normal–CRC Gene Expression Analysis Reveals Sex-Related Differences

**Figure 2:**
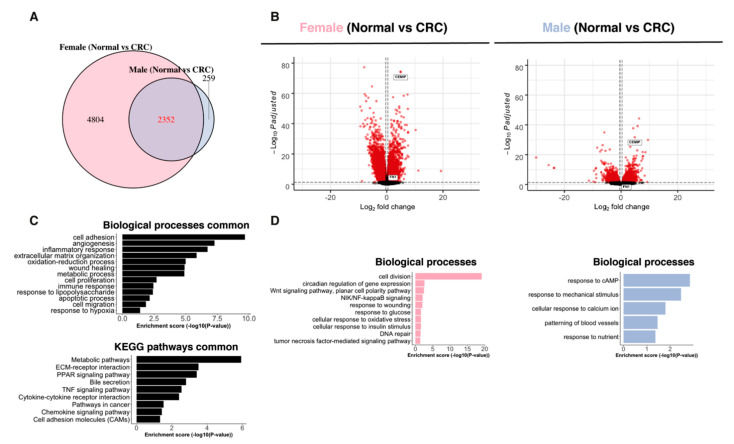
Sex-specific DEG in the tumors compared to paired normal samples. (**A**) Venn diagram comparing DEG between normal colon and CRC in females compared to males and (**B**) volcano plots of the DEG. (**C**) Biological process and KEGG pathway enrichment analysis on the DEG between normal colon and CRC in both sexes. (**D**) Biological process enrichment analysis on the sex-specific DEG between a normal colon and CRC.

Next, we compared alterations between the normal colon and CRC transcriptomes for each patient using pairwise comparisons and investigated if the sexes showed different profiles. In females, 7156 genes were differentially expressed between the paired normal colon and tumor, whereas 2611 genes were differentially expressed in males (Figure 2A). Nearly all genes regulated in male tumors (2352 out of 2611, or 90%) were also altered in female tumors. A smaller set of 259 genes appeared to have a male-specific and a larger set (4804) a female-specific tumor expression (Figure 2A). There was an equal distribution of up- and downregulated genes in both females and males (Figure 2B). The genes regulated in both male and female tumors were related to typical CRC pathways, including PPAR signaling, bile secretion, proliferation, inflammatory response, apoptosis, TNF signaling, metabolic pathways, hypoxia, and angiogenesis (Figure 2C). Female-enriched pathways included NFκB signaling, WNT signaling, cell division, DNA repair and response to glucose, and insulin (Figure 2D). In males, response to cAMP, calcium ion, nutrient, mechanical stimulus, and patterning of blood vessels were regulated (Figure 2D). Overall, females and males differed in their gene expression in both the normal colon and CRC (Figure 1B). However, the actual changes in the tumor tissue (compared to the normal tissue of the same patient) were similar to the common CRC pathways, but we also identified sex-specific differences.

### 2.4. Sex-Specific Features Independent of the Imbalanced Data

The higher numbers of DEG in females may be due to the imbalanced data (*n* = 18 for females and *n* = 6 for males). In order to exclude the effect of the imbalanced data we performed differential expression analysis on six randomly selected female tumor samples (from subtype 3 and 4) and matched normal samples in three individual runs (Figure S2A,B). The females still presented more DEG in the tumors compared to the males (Figure S2C). The common DEG between the sexes were still related to the same pathways (Figure 2C and Figure S2D) and the female-specific pathways were still related to NFκB signaling, WNT signaling, and cell division (Figure 2D and Figure S2E). The male-specific pathways were still related to response to cAMP, nutrient, and mechanical stimulus (Figure 2D and Figure S2E). Interestingly, 100% of the female-specific tumor expression in the balanced data (*n* = 6 for both females and males) overlapped with the female-specific tumor expression in the unbalanced data (*n* = 18 for females, Figure S2F). This supports that the female-specific tumor genes were not due to the imbalanced data.

### 2.5. Biomarker Discovery Revealed Common and Sex-Specific CRC Biomarkers

**Figure 3:**
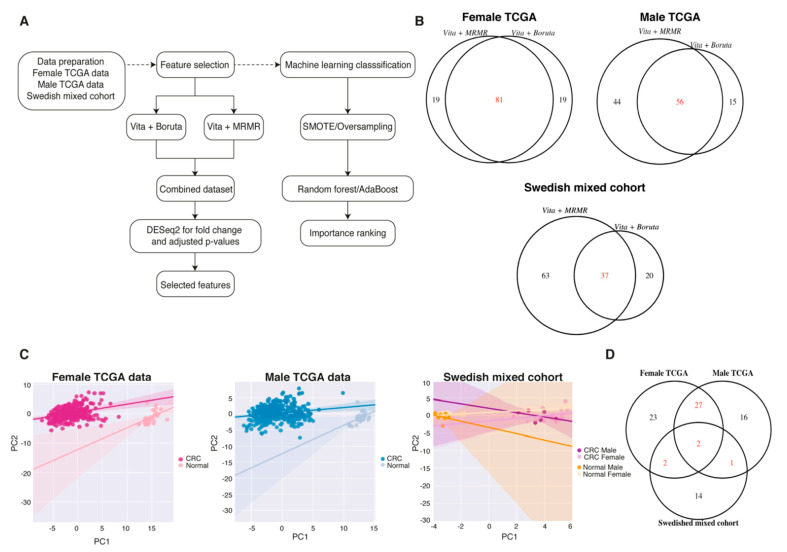
Half of the biomarkers obtained with feature selection were sex-specific. (**A**) The data-analysis pipeline for feature selection and machine learning to find the top-ranked biomarkers. (**B**) Venn diagram showing the common features selected with Vita + Boruta and Vita + MRMR. (**C**) Principal component analysis showing the separation between normal and CRC in the different datasets. (**D**) Venn diagram showing the common features between the different datasets.

To study whether sex differences impact data-driven diagnostic and prognostic biomarker discovery, we used feature selection methods separated by sex. The methods included the variable importance testing approach (Vita), minimum redundancy—maximum relevance (MRMR), and Boruta algorithm (Figure 3A). Due to the larger patient cohorts, we used The Cancer Genome Atlas (TCGA, COAD and READ) data for sex-specific biomarker discovery and combined the sexes for the Swedish cohort. With the selection criteria for Vita + Boruta and Vita + MRMR, 81, 56, and 37 features passed the selection for female TCGA, male TCGA, and Swedish mixed cohort, respectively (Figure 3B and Table 1). Next, we performed DESeq2 on the features to ensure that the selected features were significantly different between the CRC and paired normal samples (cutoff of padj < 0.05, |log_2_FC| > 2 and FPKM > 1). With the selected cutoff, 54, 46, and 19 of the features that passed the feature selection were differentially expressed for the female, male, and Swedish mixed cohorts, respectively (Table 1). The PCA plots on these differentially expressed features showed a clear separation between the non-cancerous and CRC groups in all three cohorts (Figure 3C). The biomarker discovery showed that females and males in addition to the common biomarkers also presented sex-specific ones (Figure 3D). In addition, the independent Swedish mixed cohort corroborated some biomarkers, even though the sexes were mixed, which strengthens the results (Figure 3D).

### 2.6. Top-Ranked Common and Sex-Specific Biomarkers

**Figure 4:**
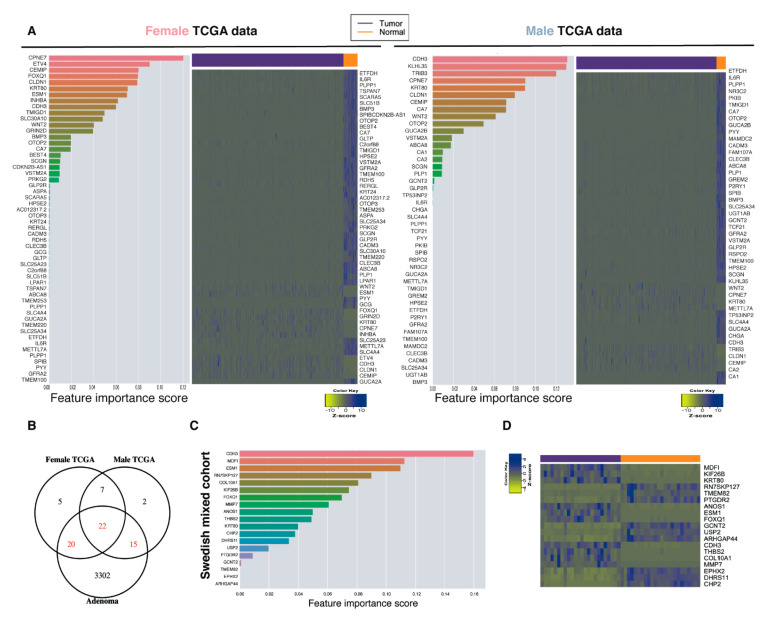
Sex-specific and common top ranked biomarkers with machine learning. (**A**) Top-ranked features with machine learning with RF for the male and female TCGA data, and heatmaps showing the expression (FPKM) of the features in a tumor and normal colon. (**B**) Venn diagram comparing the selected features in the TCGA data with an Italian cohort (GSE8671) containing 32 paired adenomas and colonic mucosa. (**C**) The top-ranked features with machine learning for our mixed cohort, and (**D**) heatmap showing the expression (FPKM) of selected features in a CRC tumor and normal colon.

**Figure 5:**
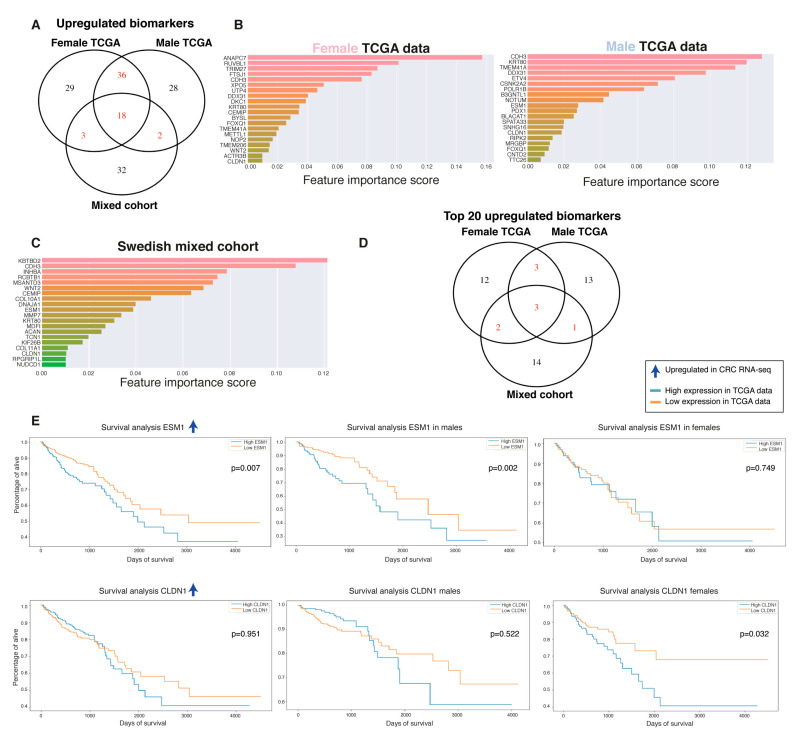
The top-ranked upregulated biomarkers *ESM1* and *CLDN1* presented a sex-specific unfavorable prognostic value. (**A**) Venn diagram illustrating the overlap of upregulated biomarkers identified with Boruta, specifically on the upregulated genes in female and male TCGA data and the Swedish mixed cohort. (**B**) The 20 top upregulated biomarkers ranked with ML using RF for female TCGA data, (**C**) male TCGA data, and (**D**) Swedish mixed cohort. (**E**) Venn diagram illustrating the overlap of the 20 top upregulated biomarkers in female and male TCGA data and the Swedish mixed cohort. (**E**) Survival analysis based on sex on TCGA data for the top-20 ranked features that significantly predicted OS.

Our data demonstrate that there are both common and sex-specific biomarkers. In order to evaluate if the best biomarkers are common or sex-specific, we performed machine-learning techniques to rank the features according to importance (Figure 3A). Random forest (RF) and adaptive boosting (AdaBoost) were used for machine learning. While RF performed best (Figure S2G), both gave similar ranked features. For the top 20 RF-ranked features, males and females presented 10 genes in common and 10 specific for each sex (Figure 4A). Next, we compared the biomarkers to an Italian cohort (GSE8671) containing 32-paired adenomas and colonic mucosa in an effort to determine if our biomarkers were regulated in the early stages of CRC and therefore could be considered as diagnostic biomarkers. The majority of the biomarkers were indeed regulated in the early stages of CRC tumorigenesis (Figure 4B). For the Swedish mixed cohort, cadherin 3 (*CDH3*) and *ESM1* were ranked as the top biomarkers and were both upregulated in the tumors (Figure 4C,D). In addition, for the biomarkers to be considered as ideal diagnostic biomarkers and potential therapeutic targets, they should present an increased expression in the diseased state. The majority of the CRC biomarkers were downregulated in the TCGA dataset (Figure 4A). In order to detect potential new therapeutic targets, we performed the feature selection on the upregulated genes with Boruta and ranked them according to their importance. Boruta detected 86, 84, and 55 important features for female TCGA, male TCGA, and the mixed Swedish cohort, respectively (Figure 5A). Reassuringly, 100% of the upregulated TCGA biomarkers, and all but one (not thrombospondin-2 (*THBS2*)) of the upregulated Swedish mixed cohort from the previous analysis remained. Eighteen biomarkers were found in all three cohorts (Figure 5A). Twenty-eight newly identified upregulated biomarkers were sex-specific (Figure 5B,D) and six of the top-20 ranked biomarkers in the Swedish mixed cohort were common in the TCGA data (Figure 5C,D). Furthermore, diagnostic biomarkers secreted into body fluids are of specific interest for screening purposes. *CEMIP*, *ESM1*, inhibin subunit beta A (*INHBA*), matrix metallopeptidase 7 (*MMP7*), and collagen type XI alpha 1 chain (*COL11A1*) were identified as biomarkers in all cohorts, and are all secreted. Furthermore, cystatin SN (*CST1*) detected in female TCGA data, transcobalamin 1 (*TCN1*) detected in the Swedish mixed cohort, and palmitoleoyl-protein carboxylesterase (*NOTUM*) detected in female and male TCGA data are also secreted and thus of potential interest as screening biomarkers.

### 2.7. Biomarkers Have Sex-Specific Prognostic Value

**Figure 6:**
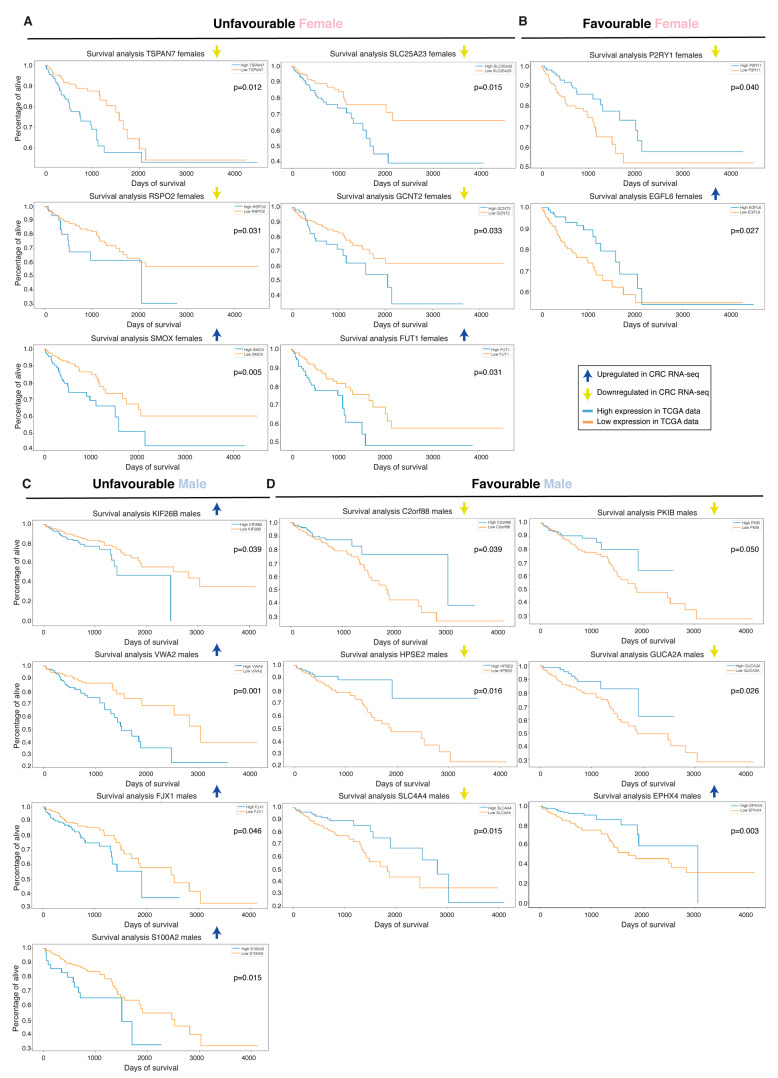
Sex-specific prognostic value of the biomarkers. Survival analysis on the features not belonging to top 20 for female and male TCGA data and for the biomarker identified in our Swedish mixed cohort that showed an unfavorable (**A**) and favorable (**B**) prognostic value in female TCGA data. (**C**) Biomarkers that showed an unfavorable (**C**) and favorable (**D**) prognostic value in male TCGA data.

Interestingly, although some of the top biomarkers were common in both sexes, the prognostic value of these could be sex-specific, and vice versa. We performed OS analysis with Kaplan–Meier plots and found that *ESM1*, an early biomarker and strong top candidate in all cohorts, showed a prognostic value when combining the sexes and had a clear unfavorable prognostic value specifically in males (Figure 5E and Table 2). *CLDN1*, a biomarker found in all three cohorts, had a clear unfavorable prognostic value for females specifically (Figure 5E and Table 2). Further down in the importance ranking lists we identified additional biomarkers with potential sex-specific prognostic values (Figure 6 and Table 2). Worth noting, solute carrier family 4 member 4 (*SLC4A4*) and kinesin family member 26B (*KIF26B*) were also significant when the sexes were combined, and showed a non-significant trend in the other sex (Figure S3A). Additional downregulated biomarkers presented a significant prognostic value when both sexes were combined but did not reach significance for either sex alone (e.g., prostaglandin D2 receptor 2/*PTGDR2*, aspartoacylase/*ASPA*, bestrophin 4/*BEST4*, and mineralocorticoid receptor/nuclear receptor subfamily 3 group C member 2/*NR3C2*; Figure S3B). None of the top-20 ranked upregulated biomarkers in CRC had prognostic value, except the previously identified biomarkers *ESM1* and *CLDN1*. However, moving down in the importance-ranking list we identified seven new biomarkers with sex-specific prognostic values (Figure 6 and Table 2). Although epidermal growth factor-like domain-containing protein 6 (*EGFL6*), *FUT1*, and four-jointed box kinase 1 (*FJX1*) presented a sex-specific prognostic value, they presented a significant prognostic value when the sexes were combined and a non-significant trend in the other sex (data not shown). Overall, our data show that females and males indeed presented a number of sex-specific top biomarkers. Even more striking is that the prognostic value of the biomarkers was highly dependent on sex, with 20 biomarkers showing a sex-specific prognostic value. This suggests that some of the diagnostic biomarkers can have a profound impact on predicting CRC prognosis when sex is taken into account, and our results indicate that sex is an important factor when evaluating CRC biomarkers.

## 3. Discussion

Our objective with this study was to evaluate if there are sex differences in the gene expression of a normal colon and CRC, and whether separating the sexes can improve the diagnostic and prognostic CRC biomarkers. Several studies have shown that there are sex differences in CRC, regarding incidence and mortality, tumor location, and mutation status [8,10,11,12]. However, very few studies consider sex differences in the analysis of tumors and biomarkers. Recently, Cai et al. demonstrated that there are sex-specific metabolic sub-phenotypes dependent on tumor location [20]. However, no studies have evaluated sex-specific CRC biomarkers at a large scale. In this study, we analyzed sex differences in the gene expression of a normal colon and CRC. Further, we analyzed if there are sex-specific diagnostic biomarkers using feature selection methods in combination with machine learning with RF to rank the selected features. To evaluate the prognostic value of the biomarkers, we performed survival analysis of the TCGA data separated by sex.

Our findings revealed significant sex differences, which, if incorporated into biomarker discovery and the clinic, could impact CRC patient outcome. First, we demonstrated sex differences in the normal colon, especially among pathways related to gluconeogenesis, bile secretion, and carbohydrate, vitamin, and lipid metabolism, all known to be dysregulated in CRC. The sex differences in the normal colon might shape the tumor characteristics and microenvironment. This can help explain the differences in male and female incidences of CRC. Estrogen menopausal hormone therapy has indeed been shown to correlate to a lower CRC incidence [21,22,23]. Although the majority of the females were in the post-menopausal state during surgery, the sex differences in the tumors may be explained by the sex differences in the normal colon. However, a larger study including normal colon tissue from both pre- and postmenopausal women would be needed to further explore the role of estrogen signaling on the colon transcriptome. Furthermore, the sex differences seen in CRC were mostly related to the immune cell response, including B-cell receptor signaling. The X chromosome contains the vast majority of immune-related genes [24], and genes that escape inactivation can influence the expression of X-linked genes and lead to sex biases in inflammatory diseases.

The sex-independent potential diagnostic biomarkers (*CLDN1*, *CEMIP*, keratin 80/*KRT80*, *CDH3*, and *ESM1*) were ranked as top features in our paired cohort. This further validates the results in a study published by Long et al., who found *CLDN1*, *CEMIP*, and *CDH3* amongst the most important features and potential diagnostic biomarkers [6]. Both ESM1 and CEMIP are secreted and can be promising CRC diagnostic biomarkers. Additionally, we found that *CLDN1* has potential as an unfavorable prognostic biomarker specifically in females. CLDN1 has previously been proposed both as a marker for CRC prognosis and as a therapeutic target [25,26], and we suggest that this may be particularly relevant for females. *ESM1* showed an unfavorable prognostic value in males. ESM1 regulates CRC cell growth and metastasis by activation of NFκB and has been shown to be of prognostic value for disease recurrence, and to correlate with a worse survival outcome [27,28]. Additionally, ESM1 is upregulated by vascular endothelial growth factor (VEGF) and is involved in hypoxia-associated angiogenesis, and further proposed as a potential therapeutic target [28]. Interestingly, we found female-specific (FtsJ RNA 2’-O-methyltransferase 1/*FTSJ1*, *CST1*, and glutamate ionotropic receptor NMDA type subunit 2D/*GRIN2D*) and male-specific (*NOTUM*, pancreatic and duodenal homeobox 1 *(PDX1*), and cyclin P/CCNP/*CNTD2*) top-ranked features, based on the TCGA data. Of note, CST1 and NOTUM are secreted and can be potential sex-specific diagnostic markers.

Moreover, *FJX1*, identified as an important biomarker in both sexes (not top 20), presented an unfavorable prognostic value specifically in males. FJX1 has also been shown to be involved in angiogenesis and associated with an unfavorable prognosis of CRC [29]. The common sex biomarker *GUCA2A* was downregulated in CRC in both sexes and showed a favorable prognostic value in males. GUCA2A mRNA and protein loss is among the most common gene losses in CRC, occurring in more than 85% of tumors [30], and has been suggested as a marker for poor prognosis [31]. GUCA2A is a peptide hormone and endogenous ligand for the guanylate cyclase 2C (GUCY2C) receptor. The loss of the GUCY2C signaling cascade due to GUCA2A downregulation promotes tumorigenesis [32]. Ligand replacement therapy to reactivate GUCY2C has been approved by the FDA or entered clinical trials [33]. Such interventions, however, relies on a maintained expression of GUCY2C. This suggests that GUCA2A can be a promising diagnostic biomarker in both sexes and may, together with the expression of GUCY2C, have a therapeutic value. Furthermore, the common sex upregulated biomarker S100 calcium-binding protein A2 (*S100A2*) was associated with an unfavorable prognostic value specifically in males. S100A2 has been shown to reprogram glycolysis and induce proliferation in CRC, and suggested as a therapeutic target [34]. High expression of S100A2 has been shown to correlate to a worse CRC OS [35].

Overall, in this study, we identified sex differences in the normal transcriptome, which may explain the sex differences in CRC susceptibility. Furthermore, we validated the previously proposed sex-independent diagnostic biomarkers *CLDN1, CEMIP*, and *CDH3* and propose new potential biomarkers. Interestingly, we did not find a single significant biomarker showing a prognostic value independent of sex, while we identified 20 diagnostic features with a sex-specific prognostic value, in particular, *ESM1*, *GUCA2A*, *FJX1*, and *S100A2* for males and *CLDN1* for females. Importantly, our study highlights the need to take sex into account in CRC research, which may improve CRC mortality.

## 4. Materials and Methods

### Patients and Samples

Clinical samples (colorectal tumors and matched noncancerous adjacent tissue) were collected from patients (*n* = 24, 18 women and 6 men) undergoing surgery in Stockholm after informed consent. The study was approved by the regional ethical review board in Stockholm (2016/957-31 and 2017/742-32). In addition, gene expression for 641 (299 women and 342 men) colorectal cancer (CRC) and 51 (28 women and 23 men) noncancerous mucosal tissues were downloaded from TCGA. The COAD and READ data were combined, the data were downloaded on 31st of January 2019, and the bioconductor package from R (Rversion 3.6.1) via the NCI Genomic Data Commons (GDC) data portal was used (TCGAbiolinks version 3.8). The molecular subtypes were determined on the Swedish cohort based on the status of the MSI, *BRAF-*, and *KRAS* mutations. The MSI status was determined using MSIsensor [36] and the *BRAF-* and *KRAS* mutation status was analyzed using the integrative genomics viewer (Broad Institute, Cambridge, MA, USA, version 2.5.2) [37]. A detailed description of the RNA isolation, quantitative PCR, gene expression analysis, feature selection, machine learning classification and overall survival analysis can be found online in the Supplementary Materials.

## Figures and Tables

**Table 1:** Number of features that passed the selection criteria for female and male TCGA data, and for the Swedish mixed cohort.

Cohort	Original Feature Numbers	Feature Selection Methods	Selected Features	Features in Common	Differentially Expressed Features
Female TCGA data	56,719	Vita + Boruta	100	81	54
		Vita + MRMR	100		
Male TCGA data	56,719	Vita + Boruta	71	56	46
		Vita + MRMR	100		
Swedish mixed	63,678	Vita + Boruta	57	37	19
		Vita + MRMR	100		

**Table 2:** Prognostic biomarkers identified in the TCGA and our Swedish cohorts.

Biomarker	Cohort ^1^	Rank ^2^	Regulation ^3^	Prognostic Value ^4^
*ESM1*	All	Top20	Up	Unfavorable in males
*CLDN1*	All	Top20	Up	Unfavorable in females
*TSPAN7*	Female TCGA	39	Down	Unfavorable in females
*SLC25A23*	Female TCGA	35	Down	Unfavorable in females
*C2orf88*	Female TCGA	36	Down	Favorable in males
*PKIB*	Male TCGA	27	Down	Favorable in males
*P2RYI*	Male TCGA	37	Down	Favorable in females
*RSPO2*	Male TCGA	29	Down	Unfavorable in females
*GCNT2*	Male TCGA and Swedish	Top20	Down	Unfavorable in females
*HPSE2*	TCGA	35 M and 25 F	Down	Favorable in males
*GUCA2A*	TCGA	31 M and 44 F	Down	Favorable in males
*SLC4A4*	TCGA	23 M and 43 F	Down	Favorable in males
*KIF26B*	Swedish	6	Up	Unfavorable in males
*PTGDR2*	Swedish	15	Down	Favorable in males and females (combined)
*ASPA*	Female TCGA	23	Down	Unfavorable in males and females (combined)
*BEST4*	Female TCGA	17	Down	Favorable in males and females (combined)
*NR3C2*	Male TCGA	30	Down	Favorable in males and females (combined)
*SMOX*	Male TCGA	37	Up	Unfavorable in females
*FUT1*	All	38 M, 78 F and 27 S	Up	Unfavorable in females
*EGFL6*	Female TCGA	27	Up	Favorable in females
*VWA2*	Male TCGA	31	Up	Unfavorable in males
*FJX1*	TCGA	67 M and 83 F	Up	Unfavorable in males
*S100A2*	TCGA	64 M and 45 F	Up	Unfavorable in males
*EPHX4*	Female TCGA	75	Up	Favorable in females

^1^ The cohort the biomarker was identified in. ^2^ The rank of the biomarker after importance ranking with machine learning. ^3^ Whether the biomarker was up- or downregulated (in the tumor and specified cohort). ^4^ Whether the biomarker correlated to a favorable or unfavorable prognostic value when highly expressed in males, females, or when both sexes were combined.

## Data Availability

The datasets used and/or analyzed during the current study are available from the corresponding author on reasonable request.

## Supplementary Materials

The following are available online at https://www.mdpi.com/1422-0067/22/3/1354/s1, Supplementary Material and Methods, Figure S1. Sex differences in the normal colon and CRC transcriptome independent of tumor location and molecular subtypes, Figure S2. Sex-specific features in tumors compared to paired normal not due to the imbalanced data, Figure S3. Overall survival analysis of the biomarkers, Table S1. Distribution of molecular subtypes, and Table S2: Upregulated biomarkers selected with Boruta.
