# Supplementary material for: The Importance of Sex in the Discovery of Colorectal Cancer Prognostic Biomarkers
Source: Int J Mol Sci. 2021 Jan 29;22(3):1354. doi: 10.3390/ijms22031354 (PMC7866425; doi:10.3390/ijms22031354)
Supplement: Supplementary file 1 [file ijms-22-01354-s001.pdf]

Supplementary information for

The importance of sex in colorectal cancer biomarker discovery

Linnea Hases, Ahmed Ibrahim, Xinsong Chen, Yanghong Liu, Madeleine Birgersson, Johan Hartman, Cecilia Williams

Corresponding author: Cecilia Williams
Email: cecilia.williams@scilifelab.se

This PDF file includes:

Material and methods
Supplementary Fig. 1. Sex differences in the normal colon and CRC transcriptome independent of tumor location and molecular subtypes.
Supplementary Fig. 2. Sex-specific features in tumors compared to paired normal not due to the imbalanced data.
Supplementary Fig. 3. Overall survival analysis of the biomarkers.
Supplementary Table 1. Distribution of molecular subtypes.
Supplementary Table 2: Upregulated biomarkers selected with Boruta.

Material and methods

RNA isolation and quantitative PCR

Frozen CRC tissue and paired noncancerous adjacent tissue stored in RNAlater were homogenized with a tissue lyser (Qiagen, Chatsworth, CA). RNA was isolated with Qiazol and purified using AllPrep DNA/RNA/Protein Mini Kit (Qiagen, Chatsworth, CA) according to the standard protocol and on-column DNAse treatment was used. Quantitative and qualitative analyses of the RNA were performed with NanoDrop 1000 spectrophotometer and Agilent 2200 Tapestation, respectively (Agilent Technologies, Palo Alto, CA). One ug RNA was reverse transcribed using iScript cDNA synthesis kit (Bio-Rad) and 10 ng of cDNA was used for the qPCR reaction in the CFX96 Touch System (Bio-Rad), with iTaq universal SYBR Green supermix (Bio-Rad) according to the manufacturer protocol. Samples were run in duplicates and the relative gene expression was calculated as the mean per group using the ΔΔCt method, normalized to the geometric mean of two reference genes (*ARHGDIA* and *ALAS1*). Paired t-test was used for comparison between paired normal and CRC specimens. A p-value <0.05 was considered statistically significant (* $p<0.05$, ** $p<0.01$).

Gene expression analysis

RNA-seq of clinical samples was performed at Sweden's National Genomics Infrastructure (NGI). Library preparation was done with Illumina RiboZero and sequenced with Illumina NovaSeq600. At least 20M 51bp paired-end reads were generated for each sample. Reads were mapped against the human genome (GRCh37) using STAR. FeatureCounts and StringTie were used to generate gene counts and FPKM values. Principal component analysis (PCA) on the gene expression (log-transformed) was used for data visualization. The R package DESeq2 (version 1.24.0) was used for differential expression analysis with raw counts as input and the Benjamini-Hochberg procedure was used to estimate FDR. Genes were considered as significantly differentially expressed if p adjusted<0.05 and log2FC>|2| and biomarkers if p adjusted<0.05 and log2FC>|2|. Gene enrichment analysis for the biological function was performed with DAVID bioinformatics website.

Feature selection methods

The Vita algorithm [17] was the first to be implemented using the vita package (version 1.0.0) in R to reduce the size of the features (threshold for p values of 0), combined with either Boruta (version 7.0.0) [18] using the boruta package or the minimum redundancy – maximum relevance (MRMR) using the mRMR package (version 2.1.0) [19], with threshold for pvalues<0.01. FPKM values were used as input. Vita is a tree-based method that randomly splits the data into two subsets of equal size, and two RFs are trained on the two subsets. Feature importance is estimated based on the other independent subset and the final importance is calculated by an average of the two scores for each feature. P-values are calculated based on the empirical distribution. Boruta is a wrapper method based on the RF classification algorithm. It creates shadow features by replicating and random shuffling of the data. The shadow dataset is attached to the original data and a RF is trained on the dataset. If the importance score of the original data is higher than the importance score of the shadow features, then the feature is considered important. mRMR

is a filter method, which selects features with high correlation with the output (i.e. relevance) and low correlation between themselves (i.e. redundancy). The features are selected one by one by maximizing the relevance and minimizing the redundancy.

Machine learning classification

Machine learning was performed in python (version 3.6.4). RF or adaptive boosting (AdaBoost) was used for classification modeling, to keep consistency with tree-based feature selection algorithms. The biomarkers obtained from the feature selection were used as input for machine learning to rank the features according to their importance. Rlog counts were used as input for the machine learning. One-third of the data was used to train the model and the rest of the data was used to test the model. The number of estimators was set to 100. Synthetic minority oversampling technique (SMOTE) [20] or randomly oversampling was used on the imbalanced TCGA data before classification. SMOTE works by drawing lines between existing minority samples in space and creates new samples randomly along those lines. Randomly oversampling work by randomly duplicating minority samples. Four different combinations were used and the accuracy, precision, recall, and AUC were recorded for each combination.

Survival analysis

COADREAD data from TCGA was used for the survival analysis, including 275 female and 322 male patients. The FPKM values were scaled and mean-centered before used for survival analysis. Python (version 3.6.4) was used for the survival analysis with FPKM and living days used as input, high expression with scale and mean-centered FPKM values was set to above zero and low expression was set to below zero, and Kaplan-Meier curves plotted. The significance was tested with log-rank test.

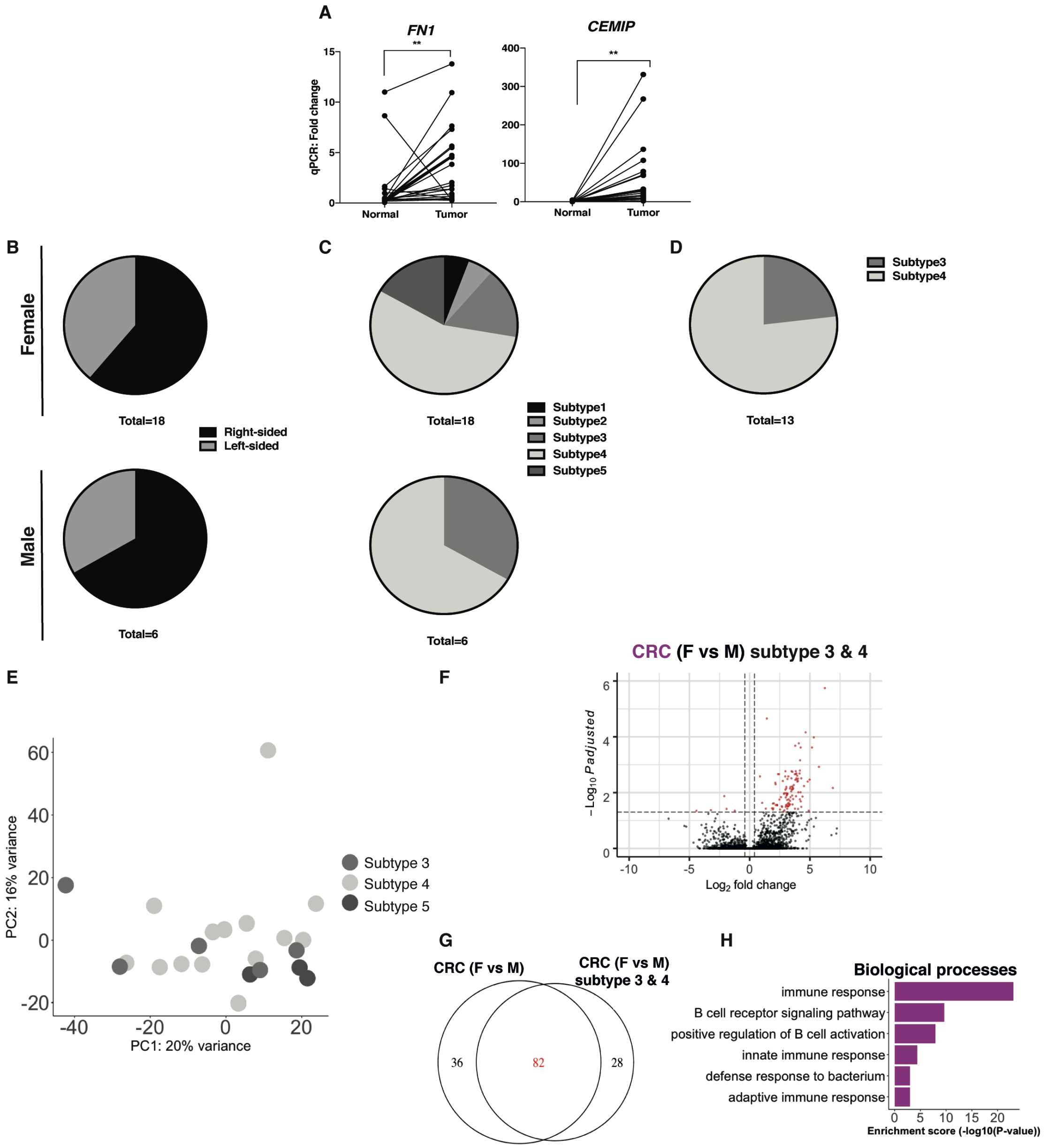

Figure S1: Sex differences in the normal colon and CRC transcriptome independent of tumor location and molecular subtypes. (A) qPCR confirmation of CRC upregulation of *FN1* and *CEMIP* in the Swedish paired normal and CRC cohort. (n=22, Student's paired t-test, * p<0.05, ** p<0.01). **(B)** Distribution of left- and right-sided tumors and molecular subtypes **(C)** in our Swedish cohort. **(D)** The distribution of Subtype 3 and 4 in females when the rest of the subtypes were removed to match the male data. **(E)** PCA plot of Subtype3-5 (Subtype1-2 were only found in one patient). **(F)** Volcano plot of the DEG between the sexes in the tumor when only subtype3-4 were included. **(F)** Venn diagram showing the overlap between the new and old analysis (including subtype3-4 or including all subtypes). **(G)** Biological processes of the common genes in the new and old analysis.

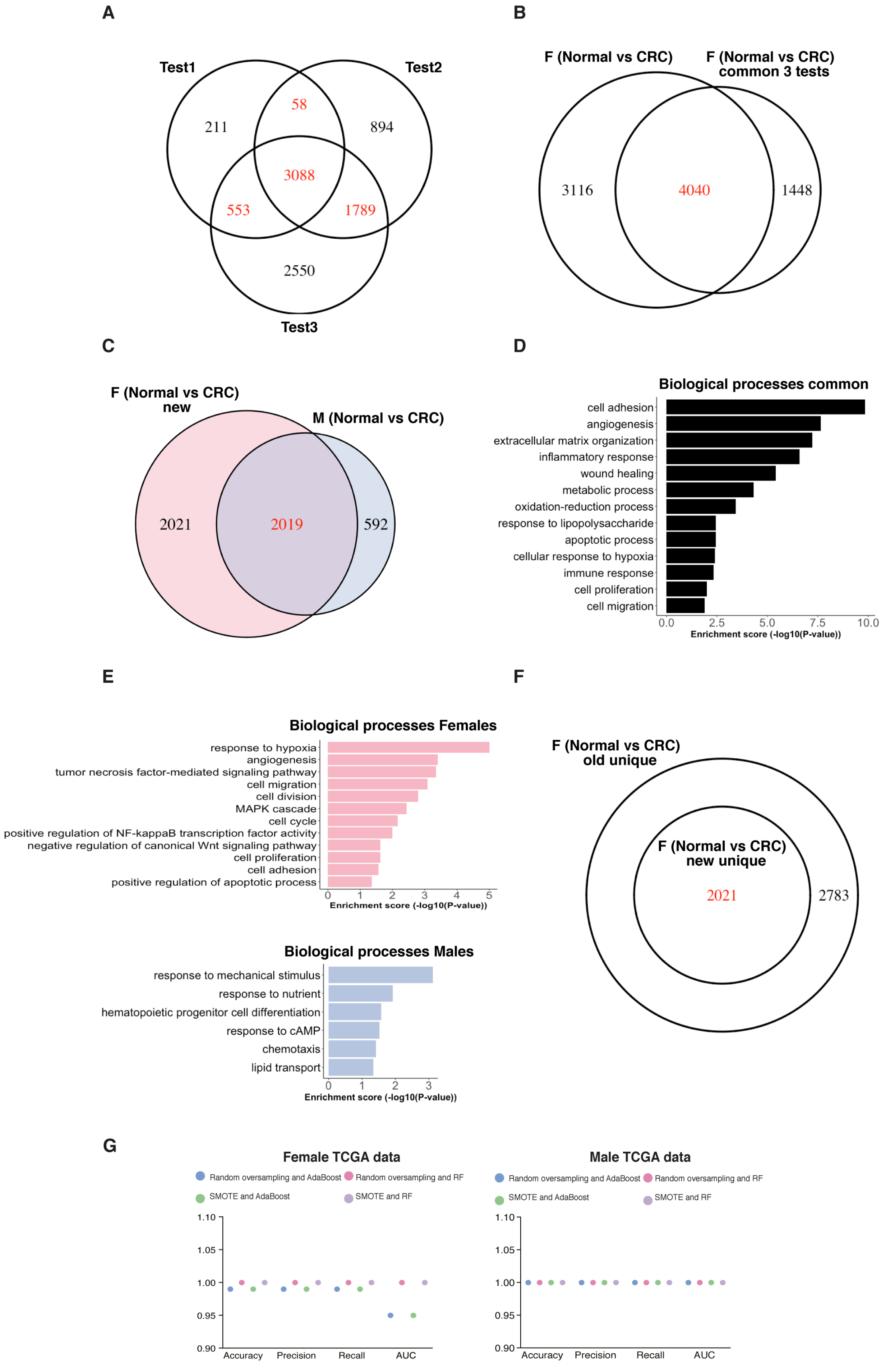

A
Test1
Test2
Test3
211
58
894
3088
553
1789
2550
B
F (Normal vs CRC)
F (Normal vs CRC) common 3 tests
3116
4040
1448
C
F (Normal vs CRC) new
M (Normal vs CRC)
2021
2019
592
D
Biological processes common
cell adhesion
angiogenesis
extracellular matrix organization
inflammatory response
wound healing
metabolic process
oxidation-reduction process
response to lipopolysaccharide
apoptotic process
cellular response to hypoxia
immune response
cell proliferation
cell migration
0.0 2.5 5.0 7.5 10.0
Enrichment score (-log10(P-value))
E
Biological processes Females
response to hypoxia
angiogenesis
tumor necrosis factor-mediated signaling pathway
cell migration
cell division
MAPK cascade
cell cycle
positive regulation of NF-kappaB transcription factor activity
negative regulation of canonical Wnt signaling pathway
cell proliferation
cell adhesion
positive regulation of apoptotic process
0 1 2 3 4 5
Enrichment score (-log10(P-value))
Biological processes Males
response to mechanical stimulus
response to nutrient
hematopoietic progenitor cell differentiation
response to cAMP
chemotaxis
lipid transport
0 1 2 3
Enrichment score (-log10(P-value))
F
F (Normal vs CRC) old unique
F (Normal vs CRC) new unique
2021
2783
G
Female TCGA data
Male TCGA data
Random oversampling and AdaBoost
Random oversampling and RF
SMOTE and AdaBoost
SMOTE and RF
1.10
1.05
1.00
0.95
0.90
Accuracy
Precision
Recall
AUC

Figure S2: Sex-specific features in tumors compared to paired normal not due to the imbalanced data. (A) Venn diagram showing the overlap of thee differentially expression analysis from 6 random female samples from subtype3-4 in paired normal and CRC samples. **(B)** Venn diagram showing the overlap of the common DEG in at least two of the three random runs (in red, 6 female samples from subtype3-4) and the original DEG in female-paired normal and CRC samples (all 18 female samples). **(C)** Venn diagram showing the overlap between the common 4040 DEG between the new and old analysis with the DEG in male-paired normal and CRC samples. Biological processes of the common **(D)** and the sex-unique **(E)** DEG in paired normal and CRC samples. **(F)** Venn diagram showing the overlap between the female-unique DEG in the old (all 18 female samples) and new analysis (6 random female samples from subtype3-4). **(G)** Accuracy, prediction, recall and AUC for random oversampling and AdaBoost; random oversampling and RF; SMOTE and AdaBoost; and SMOTE and RF for female and male TCGA data respectively.

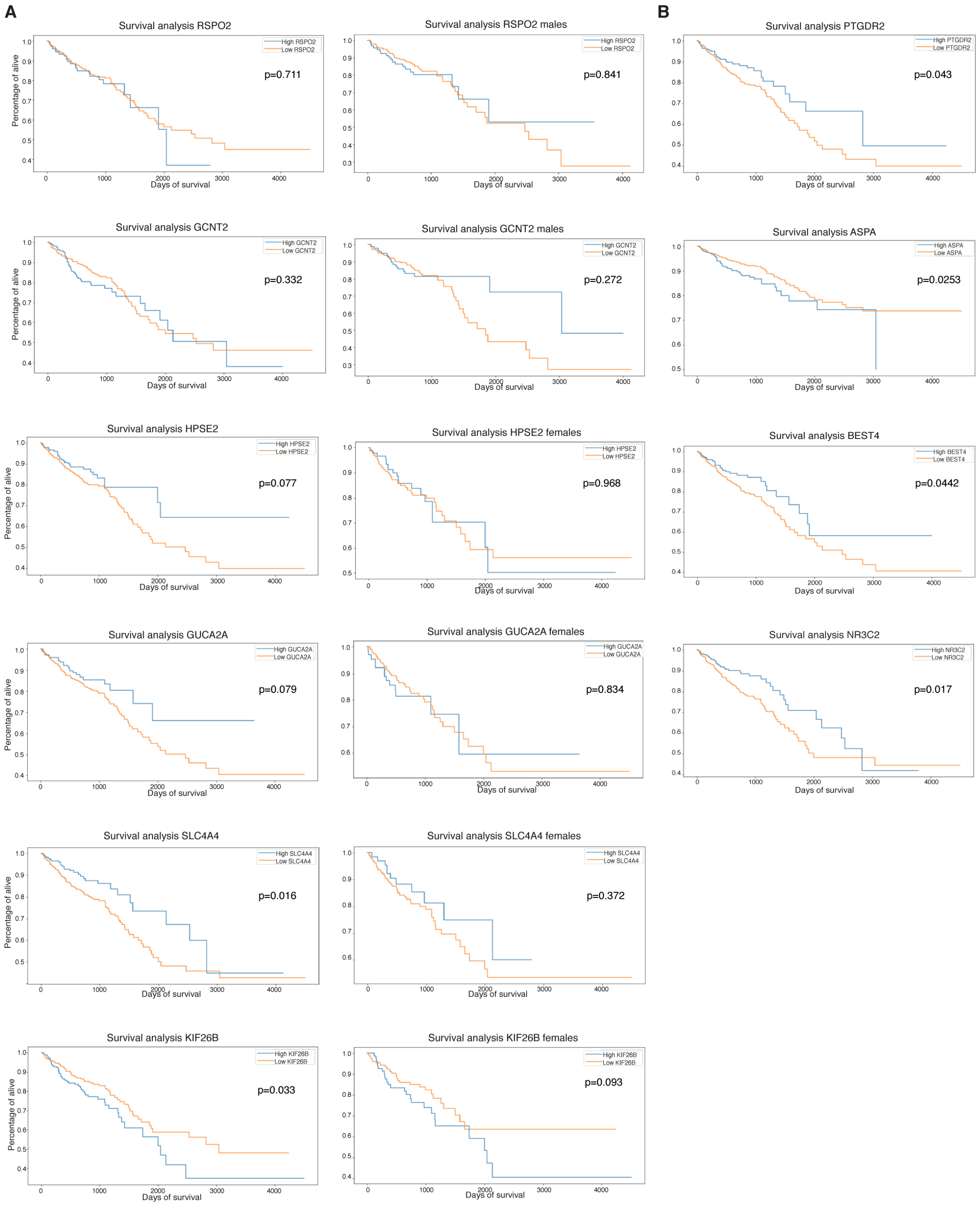

Figure S3: Overall survival analysis of the biomarkers. (A) Kaplan-Meier overall survival analysis based on sex and combined sexes from TCGA data for the prognostic biomarkers. This figure present the non-significant sex whereas the significant sex can be found in Fig. 6. **(B)** Overall survival analysis based on combined sex TCGA data. These genes did not show a significant sex-specific overall survival, but presented a significant prognostic value when both sexes were combined.

Table S1: Distribution of molecular subtypes. Percentage of CRC molecular subtype 1-5 in our clinical data correlates well to what has previously been published.

CRC subtype	MMR	CIMP	BRAF	KRAS	Our clinical data [%]	Phipps AI et al. [%]
Subtype 1	MSI	+	+	-	4	7
Subtype 2	MSS	+	+	-	4	4
Subtype 3	MSS	-	-	+	21	26
Subtype 4	MSS	-	-	-	58	47
Subtype 5	MSI	-	-	-	13	4

Table 2: Upregulated biomarkers selected with Boruta. Not belonging to the top 20 most important features ranked using ML with RF.

Rank	Female TCGA	Male TCGA	Swedish mixed
21	*ZSWIM4*	*CPNE7*	*FOXQ1*
22	*CST1*	*NKRF*	*CTHRC1*
23	*CBX2*	*TOMM34*	*SLC39A10*
24	*CD3EAP*	*CBX8*	*ANOS1*
25	*SNHG15*	*SLC39A10*	*RIPK2*
26	*ESM1*	*MMP11*	*GPR180*
27	*STRA6*	*EGFL6*	*FUT1*
28	*CBX8*	*MMP7*	*SALL4*
29	*CASC19*	*COL11A1*	*RAB36*
30	*ETV4*	*CDC25B*	*TMEM158*
31	*SALL4*	*VWA2*	*GRINA*
32	*ENC1*	*WNT2*	*ETV4*
33	*COL11A1*	*GTF2IRD1*	*DUSP14*
34	*SLC39A10*	*CBX4*	*PLAU*
35	*INHBA*	*SEC14L2*	*ARNTL2*
36	*PLEKHN1*	*LRP8*	*ADAMTS6*
37	*KLHL35*	*SMOX*	*NFE2L3*
38	*MDFI*	*PPM1H*	*FAM89A*
39	*KLK6*	*STRA6*	*PACC1*
40	*STRIP2*	*CEP72*	*MIR4435-2HG*
41	*DUSP14*	*NFE2L3*	*FXYD5*
42	*CPNE7*	*CCND1*	*ANGPT2*
43	*CEP72*	*CELSR3*	*CDH11*
44	*ARNTL2*	*ARNTL2*	*GTF3A*
45	*S100A2*	*GRIN2D*	*PLS3*
46	*CRNDE*	*MDFI*	*JADE3*
47	*SLC6A6*	*TMEM206*	*TIMP1*
48	*LRP8*	*CEMIP*	*PHLDA1*
49	*MMP11*	*SALL4*	*HECW2*
50	*TEAD4*	*ATP11A*	*GRHL1*
51	*SLC7A5*	*OSBPL3*	*AJUBA*
52	*TRIB3*	*TRIP13*	*PODXL*
53	*CDC25B*	*SLCO4A1*	*CRNDE*
54	*SLCO4A1*	*TRIB3*	*TGFBI*
55	*IQANK1*	*HILPDA*	*SNAI1*
56	*ATP11A*	*VEGFA*	
57	*TRIP13*	*FUT1*	
58	*LINC00659*	*MAFG-AS1*	
59	*AL109615.3*	*CASC19*	
60	*PVT1*	*PVT1*	
61	*NFE2L3*	*SNHG15*	
62	*MAFG-AS1*	*IQANK1*	
63	*GTF2IRD1*	*TEAD4*	
64	*TNFRSF12A*	*S100A2*	
65	*GRIN2D*	*PLEKHN1*	
66	*NOTUM*	*C6ORF223*	
67	*PPM1H*	*FJX1*	
68	*ACAN*	*ZFAS1*	
69	*EPOP*	*UBE2C*	
70	*PHLDA1*	*SPTBN2*	
71	*SIM2*	*MTHFD1L*	
72	*MMP7*	*MIR4435-2HG*	
73	*HILPDA*	*ENC1*	
74	*APLN*	*MSX1*	
75	*EPHX4*	*KLHL35*	
76	*SPTBN2*	*ZC3HAV1L*	
77	*AJUBA*	*EPOP*	
78	*FUT1*	*SLC6A6*	
79	*FOSL1*	*PRR7*	
80	*B3GNTL1*	*ULBP2*	
81	*KIAA1549*	*AJUBA*	
82	*MTHFD1L*	*CSE1L*	
83	*FJX1*	*INHBA*	
84	*MAPK15*	*PRSS22*	
85	*C6orf223*		
86	*PRSS22*		
